# Sprouty in Tumors of the Nervous System

**DOI:** 10.3390/ijms27010377

**Published:** 2025-12-29

**Authors:** Petra Obexer, Barbara Hausott

**Affiliations:** 1 Department of Pediatrics II, Medical University of Innsbruck, 6020 Innsbruck, Austria; petra.obexer@i-med.ac.at; 2 Institute of Neuroanatomy, Medical University of Innsbruck, 6020 Innsbruck, Austria

**Keywords:** Sprouty, glioblastoma, neuroblastoma, survival, proliferation, microRNA

## Abstract

The Sprouty (SPRY) proteins are evolutionarily conserved modulators of growth factor-induced signaling pathways. The four different SPRY isoforms (SPRY1-4) are implicated in different types of cancer, acting as oncogenes or tumor suppressors depending on the SPRY isoform and the malignancy. Despite being tumor suppressors in many types of cancer, SPRY1 is an oncogene in rhabdomyosarcoma, SPRY2 in colorectal cancer, and SPRY4 in gastric cancer. In this review, we summarize the current literature about the functions of SPRY1-4 in glioblastoma (GB) and neuroblastoma (NB). To further delineate the effects of SPRY1-4 in the tumorigenesis of the nervous system, we analyzed the association of SPRY1-4 with the overall and event/progression-free survival of patients with pediatric and adult glioma, GB, and NB using public datasets. Together, there is evidence that SPRY1 and -2 are oncogenes in GB, whereas the role of SPRY3 and -4 in GB is not well defined. In NB, SPRY2 acts as a tumor suppressor, whereas the effects of SPRY1, -3, and -4 in NB have not been investigated so far, although the survival analysis revealed increased survival of NB patients with low SPRY3 levels in different datasets. Thus, this review demonstrates the requirement for further studies about the functions of the SPRY proteins in tumors of the nervous system to define their clinical relevance as potential therapeutic targets in the future.

## 1. Introduction

The central nervous system (CNS) consists of the brain and the spinal cord, and the peripheral nervous system (PNS) is made up of the peripheral nerves and ganglia. The term ‘brain cancer’ summarizes multiple subtypes of tumors originating from different tissues, such as glial cells and/or neurons of the CNS. According to the fifth edition of the World Health Organization classification of tumors of the CNS (WHO CNS5), published in 2021, the classification of tumors of the CNS has undergone major changes due to advances in molecular genetics. CNS tumors now include different types of gliomas, glioneuronal and neuronal tumors, choroid plexus tumors, embryonal tumors, pineal tumors, cranial/paraspinal nerve tumors, meningiomas, mesenchymal (non-meningothelial) tumors, melanocytic tumors, hematolymphoid tumors of the CNS, germ cell tumors, tumors of the sellar region, and metastases to the CNS. The taxonomy and nomenclature of all CNS tumors became more standardized, and new tumor types were added. Histopathological and molecular features are now included in tumor grading, which changed from Roman grade numbers to Arabic numbers [1,2]. Neuroblastoma (NB) is an aggressive childhood tumor that originates from the neural crest during the embryogenic development of the PNS and the adrenal gland. NB occurs most frequently in the adrenal glands but also along the sympathetic nervous system [3]. Staging and risk classification depend on clinical and imaging factors according to the International Neuroblastoma Risk Group Staging System (INRGSS) [4] or the revised classification system, which incorporates segmental chromosome aberrations (SCAs) as an additional genomic biomarker [5].

### 1.1. Glioblastoma

Gliomas are the most prevalent types of brain tumors, which arise from the glial cell types astrocytes, oligodendrocytes, ependymal cells, and microglia. According to the WHO CNS5, gliomas, glioneuronal tumors, and neuronal tumors are grouped into six families of adult-type diffuse gliomas, pediatric-type diffuse low- and high-grade gliomas, circumscribed astrocytic gliomas, glioneuronal/neuronal tumors, and ependymomas. Adult-type diffuse gliomas are grouped as those with isocitrate dehydrogenase (IDH) mutations (IDH-mutant) and those without IDH mutations (IDH-wildtype), and they constitute the three categories of IDH-mutant astrocytoma (grade 2, 3, or 4), IDH-mutant and 1p/19q-codeleted oligodendroglioma (grade 2 or 3), and IDH-wildtype glioblastoma (GB; grade 4). GB is defined as IDH-wildtype diffuse astrocytic glioma with at least one of the following three criteria: gain of whole chromosome 7 and loss of whole chromosome 10 (+7/−10), telomerase reverse transcriptase (TERT) promoter mutations, and epidermal growth factor receptor (EGFR) amplification. The previously common term ‘glioblastoma multiforme’ (GBM) is no longer in use [1,2,6]. GB is the most prevalent (50.1%), aggressive, and lethal form of brain tumors. Its incidence increases with age, whereas it is extremely rare in a pediatric population [7]. The standard treatment for GB, according to the Stupp protocol [8], has not changed substantially in decades and includes a maximal surgical resection along with temozolomide chemotherapy and radiotherapy. However, even with this standard therapy, the median overall survival is less than 18 months, and only 5.8% of GB patients survive for more than five years [6,9].

Receptor tyrosine kinases (RTKs) are involved in the tumorigenesis of GB. EGFR alterations are present in 50–60% of GBs, including overexpression, gene amplification, and mutation [10,11,12]. Platelet-derived growth factor receptor alpha (PDGFRα) is the second most frequently amplified RTK in GB [13]. Although fibroblast growth factor receptor (FGFR) mutations, amplifications, and/or fusions are relatively rare in GB, FGFR1 expression is increased in GB compared to normal brain tissue, and its expression correlates with the grade of malignancy. High expression of FGFR1, but not of FGFR2-4, correlates with reduced survival of GB patients [14,15,16,17]. Oncogenic fusions between the tyrosine kinase domains of FGFR1 and FGFR3 with the transforming acidic coiled-coil (TACC) coding domains of TACC1 and TACC3, respectively, occur in about 3% of GB patients and result in ligand-independent FGFR activation [18]. Interestingly, FGFR1-TACC1 and FGFR3-TACC3 fusions have only been detected in IDH-wildtype GB but not in IDH1/2-mutant glioma, indicating the relevance of FGFR-TACC fusions in the malignancy [19].

Despite the important roles of RTKs in GB, the clinical effects of most RTK inhibitors for the treatment of GB are disappointing [6]. Interestingly, the treatment of GB cells with inhibitors for EGFR and hepatocyte growth factor receptor (MET) induces fibroblast growth factor (FGF) signaling. Thus, co-treatment with a combination of three inhibitors for EGFR, MET, and FGFR reduces the tumor size of subcutaneous and intracranial GB xenografts in mice to a greater extent than treatment with two inhibitors for EGFR and MET [20]. Similarly, a combination of PDGFR inhibitors with inhibitors for either Erb-B2 receptor tyrosine kinase 3 (ERBB3) or insulin-like growth factor 1 receptor (IGF1R) suppresses the growth of GB cells more potently than each inhibitor alone [21]. Since GB is characterized by the mosaic expression of different RTKs, such as EGFR, PDGFRα, and MET by neighboring cells [22,23], the combination of different RTK inhibitors or the use/development of RTK inhibitors targeting multiple RTKs may be useful. The selective FGFR1-3 inhibitor Infigratinib revealed limited efficacy in GB patients with different genetic FGFR alterations, but it had an effect on permanent disease control in patients with tumors harboring FGFR1 or FGFR3 point mutations or FGFR3-TACC3 fusions [24]. This is in line with a previous report stating that the pan-FGFR inhibitor JNJ-42756493 results in clinical improvement of patients with FGFR3-TACC3 rearrangements [19].

### 1.2. Neuroblastoma

NB is an aggressive childhood tumor with a low mutational burden that originates from precursor cells of the neural crest during embryogenesis. It accounts for 7% of all childhood cancers and is the leading cause of pediatric cancer mortality for children aged one to five years [3]. NB is characterized by a variety of genetic and biological variations, as well as by clinical heterogeneity, such as poor survival rates and treatment resistance, but also spontaneous regression [25]. NB is categorized into high-risk, intermediate-risk, and low-risk tumors. Low-risk tumors have a good prognosis and are diagnosed in children younger than 18 months. The majority of patients with high-risk NB are older than 18 months at onset and show disseminated disease. High-risk NB is responsible for a high infant mortality rate, and approximately 50% of high-risk patients relapse and demonstrate a drug-resistant disease [5,26,27,28,29]. Crucial oncogenic drivers for the development of NB are MYCN amplification (25–30%), anaplastic lymphoma kinase (ALK) mutations (8–10%), and segmental chromosomal alterations such as extra copies of the 17q chromosome arm and deletions on chromosome arms 1p and 11q [26,30]. Furthermore, mutations of genes of the rat sarcoma (RAS)/mitogen-activated protein kinase (MAPK) pathway are detected in NB [30,31]. In relapsed tumors, the mutation frequency of the ALK and RAS/MAPK pathway genes is enhanced, demonstrating that activation of this pathway mediates a more aggressive phenotype. Thus, patients with an activated RAS/MAPK pathway show reduced overall survival [32,33,34].

Different RTKs, such as the rearranged during transfection (RET) receptor, IGF1R, insulin receptor (IR), and FGFR1, are activated in NB cells [35]. High expression of RET is associated with reduced survival in NB patients, and inhibition of RET with Vandetanib or Cabozantinib decreases tumor growth in cell lines and mouse models [36,37,38]. Although alterations in the FGFR1-4 genes are rare in NB, at only about 0.2%, high expression of FGFRs correlates in those cases with a worse prognosis [39]. Higher FGFR1 expression is associated with reduced relapse-free and overall survival of NB patients, and relapsed tumors show a higher FGFR1 expression than primary NB. The FGFR1 inhibitor AZD4547 reduces the invasive capacity and colony number of NB cells overexpressing FGFR1. The somatic mutation N546K in FGFR1 mediates resistance to AZD4547 treatment through activation of the protein kinase B (PKB/Akt) and the signal transducer and activator of transcription 3 (STAT3) pathways. In cells that overexpress FGFR1 harboring the N546K mutation, the combined use of the FGFR1 inhibitor AZD4547 with the phosphoinositide 3-kinase (PI3K) inhibitor GDC0941 is necessary to decrease invasion and neurosphere formation [40]. Alterations in the PDGFR or EGFR genes are infrequent in NB, but expression of PDGFRβ is associated with a favorable prognosis [41,42]. The tropomyosin receptor kinases TrkA, TrkB, and TrkC, together with their ligands nerve growth factor (NGF), brain-derived neurotrophic factor (BDNF), neurotrophin-3 (NT3), and neurotrophin-4 (NT4), regulate growth and differentiation of neuronal cells. TrkA expression is linked to a less aggressive phenotype and improves NB patient outcomes because NGF promotes differentiation of TrkA-expressing tumors [36,43]. Tumors with expression of TrkB frequently show MYCN amplification and are aggressive and lethal, whereas TrkC is expressed predominantly in favorable NBs that lack MYCN expression [44,45]. The Trk inhibitors (CEP-751, AZ64, GNF-4256, and Lestaurtinib) have efficacy in NB cell lines and in xenograft models by reducing tumor growth and improving survival [46,47,48,49].

The standard therapy for high-risk NB patients involves surgery, high-dose chemotherapy, radiotherapy, and autologous stem cell rescue [27,28]. Tumors with ALK mutations are treated with ALK inhibitors such as Ceritinib, Alectinib, and Lorlatinib [50]. The consolidation therapy includes anti-disialoganglioside GD2 monoclonal antibodies, often in combination with retinoic acid. Despite intensive therapies, high-risk NB patients display a low survival rate, and therefore, it is crucial to develop novel therapeutic targets to improve patient survival [51].

## 2. Sprouty Proteins

The first Sprouty (SPRY) protein was discovered in drosophila (dSPRY) in 1998, revealing the inhibitory function of dSPRY in the FGF signaling pathway. In a search of the expressed sequence tag (EST) database, three human homologs of the fruit fly gene designated as hSPRY1-3 were identified [52]. The fourth mammalian SPRY homolog, hSPRY4, was later discovered in humans [53]. All four SPRY proteins are characterized by a conserved C-terminal cysteine-rich domain (CRD), a serine-rich motif (SRM), and a variable N-terminal domain containing a key tyrosine residue. Among the four SPRY isoforms, SPRY2 exhibits the highest evolutionary conservation, with the human SPRY2 showing 97%, 85%, and 51% sequence homologies in the CRD domain compared to the mouse, chicken, and drosophila proteins, respectively [52,54].

### 2.1. Regulation of Sprouty Proteins

**Figure 1:**
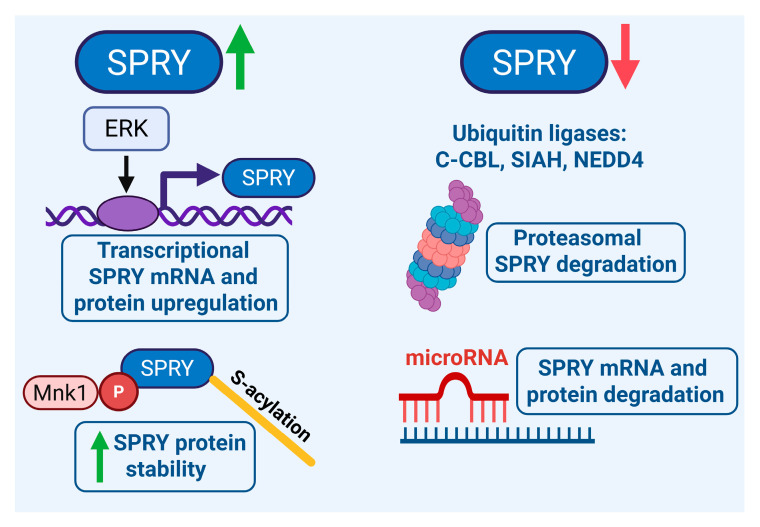
Regulation of Sprouty (SPRY) by transcription, post-translational modification, proteasomal degradation, and microRNAs (miRs). Left: Growth factor-induced activation of extracellular signal-regulated kinase (ERK) upregulates SPRY mRNA and protein. Phosphorylation by mitogen-activated protein kinase-interacting kinase 1 (Mnk1) and S-acylation increases the protein stability of SPRY. Right: The ubiquitin ligases c-casitas b-lineage lymphoma (c-CBL), seven in absentia homolog 2 (SIAH2), and neural precursor cell-expressed developmentally downregulated protein 4 (NEDD4) are involved in proteasomal degradation of SPRY proteins. Different miRs regulate SPRY mRNA and protein. Image was created in BioRender. Hausott, B. (2025) https://BioRender.com/nbsmawp.

SPRY proteins are tightly regulated on multiple levels, from transcriptional regulation to different post-translational modifications (Figure 1). On the transcriptional level, growth factor treatment and the activation of the extracellular signal-regulated kinase (ERK) pathway upregulate SPRY mRNA and proteins [55,56,57]. Reduced ERK activation decreases the SPRY2 protein, whereas enhanced activation of ERK increases the SPRY2 protein in U87 and U118 GB cells [20]. Insulin-like growth factor binding protein-2 (IGFBP2) increases SPRY1 expression in U87 GB cells through the activation of nuclear factor kappa B (NF-κB) signaling [58]. EGF stimulation increases the SPRY2 protein in patient-derived GB stem cells [59]. By contrast, the SPRY3 protein is barely regulated by serum in different GB and NB cell lines, whereas the SPRY4 protein is increased by serum in some of these cell lines [60]. SPRY2 and -4 mRNAs are increased by glial cell line-derived neurotrophic factor (GDNF) in TWG NB cells. By contrast, the mRNA levels of SPRY1 and SPRY3 are low, and their induction by GDNF is moderate in these cells. The strongest mRNA induction was observed with SPRY2 in a biphasic way, and the SPRY2 protein is also more strongly increased by GDNF than the SPRY4 protein [61]. SPRY2 is also regulated during the differentiation of a subset of NB cells [62].

The ubiquitin ligases c-casitas b-lineage lymphoma (c-CBL), seven in absentia homolog 2 (SIAH2), and neural precursor cell-expressed developmentally downregulated protein 4 (NEDD4) are implicated in the ubiquitination and proteasomal degradation of SPRY proteins [63,64,65,66,67,68,69]. Hence, SPRY2 protein reduction during the late G1 phase of the cell cycle is regulated by its proteasomal degradation by c-CBL [57]. Phosphorylation by mitogen-activated protein kinase-interacting kinase 1 (Mnk1) or S-acylation (palmitoylation) of the CRD increases the protein stability of SPRY2 [70,71]. Mnk1 phosphorylates SPRY2 on the two serines S112 and S121, and the mutation of these serine residues or the inhibition of Mnk1 increases the degradation of SPRY2 [70]. The CRD of SPRY2 is efficiently S-acylated by different S-acyltransferases, and S-acylation plays an important role in the association of proteins with various intracellular compartments. Thus, SPRY2 is associated with the plasma membrane, caveolin-1, microtubules, and endosomes [71,72,73,74,75,76]. In GB and NB cells, SPRY2 is localized in punctate structures in the cytoplasm, and fewer spots have been observed in NB cells expressing truncated SPRY2 lacking its CRD. However, the C-terminus of SPRY2 is not required for the formation of cytoplasmic spots in GB cells since SPRY2 lacking the C-terminal domain exhibits similar cytoplasmic patterns to full-length SPRY2 [77,78]. In GB cells, SPRY2 is mainly localized to the plasma membrane, early and late endosomes (lysosomes), and vimentin filaments, whereas localization to microtubules is rarely observed in these cells. However, plasma membrane localization is clearly reduced in GB cells with SPRY2 lacking the C-terminal domain as compared to full-length SPRY2, confirming the role of the CRD in the plasma membrane localization of SPRY2 [71,78].

MicroRNAs (miRs) play an important role in the tumorigenesis of GB and NB [79,80,81], and different SPRY isoforms are regulated by miRs [82,83,84]. The miRs that regulate SPRY isoforms in GB and NB are summarized in Table 1. MiR-21 is deregulated in many tumors, including GB and NB, and miR-21 has been shown to target SPRY1 and -2. The SPRY1 protein is reduced by miR-21 in neurosphere cultures from patient-derived GB cells, and anti-miR-21 increases the SPRY2 protein in GB cells. MiR-21 expression is upregulated in human GB tissue and correlates inversely with the SPRY2 protein levels [85,86,87]. Furthermore, miR-21 is upregulated in WHO CNS5 grade 1 pediatric pilocytic astrocytoma [88]. MiR-21 is also increased in NB tissue compared with matched adjacent non-tumor tissue and reduces SPRY2 protein in NB cells [89,90]. The forkhead box O3 (FOXO3) transcription factor binds to the promoter region of miR-21, thereby repressing miR-21 expression in NB cells and inhibiting the binding of miR-21 to SPRY2, which increases the SPRY2 protein [90,91]. MiR-27a and -27b are increased in tumor tissue from GB patients compared to normal brain tissue, and both miRs reduce the SPRY2 protein in GB cells [92,93]. MiR-124 reduces the SPRY1 protein in cortical neuron cultures and is involved in the proliferation and differentiation of NB cells [94,95,96]. SPRY4 mRNA and protein are downregulated by miR-1908 in U251 GB cells, and miR-1908 is upregulated in GB tissue [97]. Furthermore, RNA sequencing has identified miR-25-5p and miR-130b-5p targeting SPRY4 in GB [98].

### 2.2. Signaling Mechanisms of Sprouty Proteins

The major function of SPRY proteins is the modulation of growth factor-induced signaling pathways. Different SPRY isoforms modulate signaling of the MAPKs ERK, Jun N-terminal kinase (JNK), and p38. Furthermore, activation of phospholipase C (PLC) and Akt is influenced by SPRY proteins depending on the cell type and the growth factors involved [82,84,99]. SPRY proteins reduce signaling in response to several growth factors, including FGF, BDNF, NGF, GDNF, PDGF, and vascular endothelial growth factor (VEGF) [74,100,101,102,103,104]. In comparison to most other growth factors, EGF-induced signaling is differently controlled by SPRY. Depending on the cell type, it has either no effect, an inhibitory effect, or a signal-enhancing effect [64,74,105,106,107]. SPRY-induced sustained EGF-signaling is caused by the inhibition of EGFR endocytosis, ubiquitination, and degradation [64,108,109].

**Figure 2:**
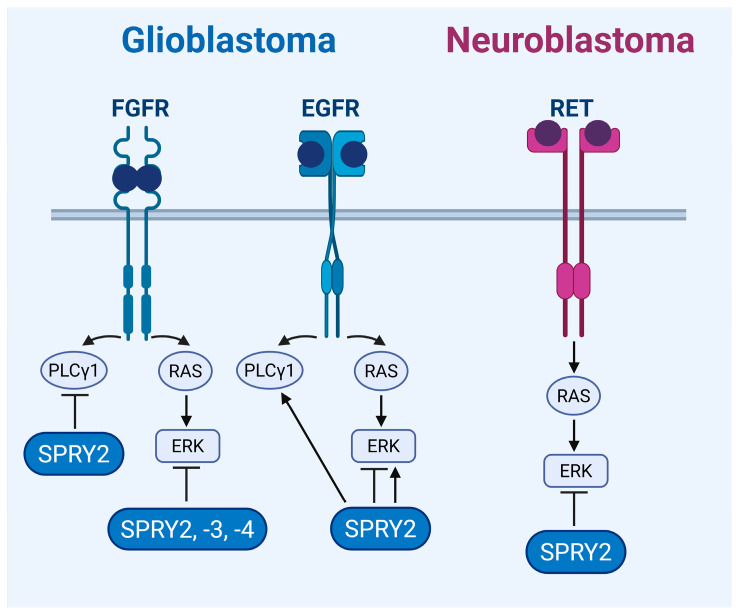
The effects of SPRY on receptor tyrosine kinase (RTK) signaling in GB and NB cells. SPRY2, -3, and -4 inhibit fibroblast growth factor receptor (FGFR)-induced ERK signaling, and SPRY2 inhibits FGFR-induced phospholipase Cγ1 (PLCγ1) activation in GB cells. SPRY2 increases or inhibits epidermal growth factor receptor (EGFR)-induced ERK signaling and enhances EGFR-induced PLCγ1 activation in GB cells. ERK signaling induced by glial cell line-derived neurotrophic factor (GDNF)/rearranged during transfection (RET) receptor is inhibited by SPRY2 in NB cells. Image was created in BioRender. Hausott, B. (2025) https://BioRender.com/bqhu406.

In the U87 GB cell line, SPRY2 knockdown increases the phosphorylation of ERK, JNK, and p38 [110]. The effects of SPRY2 knockdown on the activation of ERK and Akt in U87 cells are further increased by EGF treatment, and the same effect of SPRY2 knockdown on EGF-induced ERK and Akt activation is observed in patient-derived GB stem cells [59]. By contrast, SPRY2 knockdown has no effect on JNK activation in U251 and U118 cells, whereas p38 activation is increased in U118 but not in U251 cells. The effect of SPRY2 knockdown on p38 and JNK activation in U87 cells is regulated by a reduction in the dual-specificity mitogen-activated protein kinase phosphatases MKP-1 and MKP-5 [110]. In U251 cells overexpressing FGFR1, SPRY2 overexpression inhibits FGF2-induced activation of PLCγ1 and ERK and reduces binding of PLCγ1 to FGFR1. Knockdown of SPRY2 increases FGF2-induced activation of PLCγ1, but not of ERK, in SF126 cells overexpressing FGFR1. In U251 cells overexpressing EGFR, SPRY2 overexpression increases EGF-induced activation of PLCγ1, but not of ERK. Knockdown of SPRY2 reduces EGF-induced activation of PLCγ1 and ERK in SF126 cells overexpressing EGFR [111]. FGF2-induced phosphorylation of ERK is inhibited by overexpression of SPRY3 or -4 in U373 GB cells, with a stronger effect of SPRY4 overexpression. Serum-induced phosphorylation of ERK is impaired by overexpression of SPRY4 but not of SPRY3 in U373 cells [60] (Figure 2).

In NB TGW cells, overexpression of SPRY2 inhibits GDNF-induced ERK activation, whereas dominant-negative SPRY2 enhances GDNF-induced ERK activation [61]. The inhibitory function of SPRY2 on ERK activation in NB cells is further confirmed by the effect of FOXO3, which inhibits ERK phosphorylation via inhibition of miR-21 that increases the SPRY2 protein in vitro and in vivo [90]. In Neuro-2A NB cells, SPRY2 was identified as an interaction partner of G protein-regulated inducer of neurite outgrowth (GRIN), and SPRY2 competes with the G-protein Gα_o_ in binding to GRIN. Overexpressed GRIN increases FGF2-mediated ERK activation and reduces SPRY2 phosphorylation. GRIN-bound SPRY2 is unable to inhibit FGF2-induced ERK activation. When Gα_o_ binds to GRIN, SPRY2 is released and inhibits FGF2-induced ERK activation [112] (Figure 2).

## 3. Sprouty in Tumors of the Nervous System

### 3.1. Sprouty and Glioblastoma

**Figure 3:**
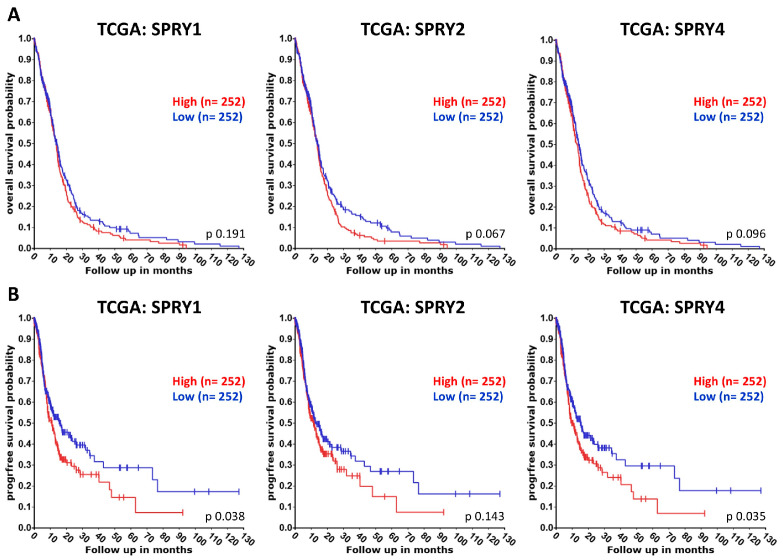
The association of the expression of SPRY1, -2, and -4 with the overall (**A**) and progression-free (**B**) survival of GB patients was evaluated using the Cancer Genome Atlas (TCGA) dataset. Overall survival of GB patients with high SPRY2 levels and progression-free survival of GB patients with high SPRY1, -2, and -4 levels are reduced.

**Figure 4:**
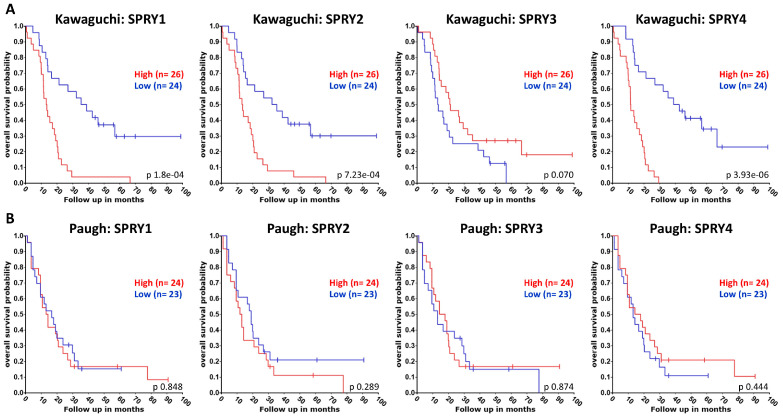
The association of the expression of SPRY1-4 with the overall survival of adult glioma patients was evaluated using Kawaguchi datasets (**A**) and of pediatric glioma patients using Paugh datasets (**B**). Overall survival of adult glioma patients with high SPRY1, -2, and -4 levels is strongly reduced, whereas it is non-significantly enhanced in patients with high SPRY3 levels. Survival of pediatric glioma patients with high SPRY2 and low SPRY4 levels is non-significantly reduced.

The four SPRY isoforms act as oncogenes or tumor suppressors depending on the SPRY isoform and the type of cancer [82,84,113]. Here, we summarize the existing literature about the effects of the four different SPRY isoforms in GB. In addition, we used the R2 genomics analysis and visualization platform (http://r2.amc.nl accessed on 31 October 2025) to evaluate the association of SPRY1-4 with the overall and progression-free survival of GB patients using datasets from the Cancer Genome Atlas (TCGA: https://www.cancer.gov/tcga accessed on 31 October 2025). Overall survival of adult glioma patients was analyzed using Kawaguchi datasets (GSE43378) and of pediatric glioma patients using Paugh datasets (GSE19578), although the sample size was smaller than that of the TCGA dataset of GB patients. Univariate Kaplan–Meier analyses were performed by setting the median expression of SPRY1-4 as the cut-off because the median is the most common measure for survival data and is insensitive to outliers [114]. The resulting survival curves and *p*-values (obtained via the log-rank test with Bonferroni correction for multiple comparison) were downloaded (Figure 3 and Figure 4).

In GB tissue, SPRY1 mRNA and protein are significantly upregulated compared to normal brain tissue or lower-grade gliomas, and high SPRY1 levels are related to reduced overall survival of GB patients using the REMBRANDT datasets [58,115,116,117]. Our analysis of the TCGA datasets revealed that high SPRY1 levels reduce the progression-free survival of GB patients but not their overall survival (Figure 3). Furthermore, the overall survival of adult glioma patients with high SPRY1 levels is strongly reduced in the Kawaguchi datasets (Figure 4A), whereas there is no difference in the overall survival of pediatric glioma patients with high SPRY1 levels in the Paugh datasets (Figure 4B). Furthermore, the expression of SPRY1 is higher in glioma stem cells than in GB cells. Downregulation of SPRY1 reduces the stemness and the self-renewal ability of glioma stem cells, which is important because self-renewal of glioma stem cells is associated with tumor recurrence and therapeutic resistance [116,118]. IGFBP2, which is upregulated in GB and associated with poor diagnosis of GB patients as well, increases SPRY1 mRNA expression in U87 GB cells, whereas IGFBP2 knockdown reduces SPRY1 in U251 GB cells. SPRY1 knockdown impairs the invasion capability of U251 cells and reduces the expression of mesenchymal markers, including N-cadherin, vimentin, Snail, and Twist1 [58]. MiR-21 is upregulated during the differentiation of GB neurosphere cultures from patient biopsies, whereas SPRY1 protein is decreased during this process. Overexpression of miR-21 promotes differentiation of GB neurosphere cultures and reduces SPRY1, suggesting that miR-21 induces differentiation by targeting SPRY1 in these cells [87].

SPRY2 mRNA expression is upregulated in GB, and the upregulation of SPRY2 correlates with the glioma grade. High SPRY2 levels are also associated with reduced GB survival rates, especially in younger patients [59,110]. Our R2 genomics analysis of the TCGA datasets reveals the same effects of high SPRY2 levels on the reduced overall and progression-free survival of GB patients, although this is not significant (Figure 3) compared to the GlioVis analysis of the TCGA datasets [59]. Furthermore, the overall survival of adult and pediatric glioma patients with high SPRY2 levels is reduced (Figure 4). Other studies found no increase in SPRY2 mRNA but reduced SPRY2 protein levels in higher malignant gliomas [86]. However, GB cell lines, primary GB cells from patients, and cultured astrocytes differ in their SPRY2 mRNA and protein levels. Among the widely used GB cell lines, SPRY2 mRNA and protein are low in U251, U1242, and T98G cells, whereas they are high in U118, U87, and SF126 cells [59]. SPRY2 knockdown reduces the proliferation of U87, U251, U118, and patient-derived primary GB cells, but it has no effect on the proliferation of astrocytes [59,110]. The excessive ERK activation induced by SPRY2 knockdown results in the premature onset of DNA replication and increased DNA damage. Thus, the mitogen-activated protein kinase (MEK) inhibitor PD98059, which inhibits ERK activation, reverses the inhibitory effect of SPRY2 knockdown on cell proliferation [59]. Furthermore, SPRY2 downregulation antagonizes the colony formation of U87, U251, and U118 GB cells in soft agar [110]. Co-inhibition of EGFR and MET transiently suppresses ERK activation and reduces SPRY2 protein in U87 cells. This is followed by activation of NF-κB, which leads to autocrine FGFR activation by increased FGF1 secretion, reactivation of ERK, and an increase in the SPRY2 protein. The inhibition of ERK reactivation or FGFR activation prevents an increase in SPRY2 protein and enhances cell death in response to EGFR and MET co-inhibition [20]. In U251 cells overexpressing FGFR1, overexpression of SPRY2 promotes proliferation and increases the resistance to cisplatin treatment. SPRY2 knockdown has no effect on the proliferation of SF126 cells overexpressing FGFR1 despite their high endogenous SPRY2 levels, but SPRY2 knockdown increases the sensitivity to cisplatin treatment in these cells [111].

The inhibitory effect of SPRY2 knockdown on tumor growth has been demonstrated in vivo using subcutaneous and intracranial mouse tumor xenograft models of U87 cells [59,110]. Treatment of mice bearing subcutaneous U87 cell xenografts with an FGFR inhibitor reduces tumor growth. The inhibitory effect on tumor growth is further enhanced by co-inhibition of FGFR, EGFR, and MET, which reduces activation of ERK and Akt. Overexpression of SPRY2 abolishes the inhibitory effect of co-inhibition of FGFR, EGFR, and MET on tumor growth despite reduced ERK and Akt activation, suggesting that additional mechanisms are involved in the tumorigenic potential of SPRY2 in GB [20]. Overexpression of SPRY2 in low tumorigenic U251 cells promotes tumor growth of subcutaneous mouse xenografts. However, overexpression of mutant SPRY2, in which S121 was replaced by alanine (SPRY2^S121A^), completely blocked the tumor formation of U251 cells in subcutaneous mouse xenografts in [59]. The S121 phosphorylation site is involved in the protein stability of SPRY2 [70], and the inhibitory effect of SPRY2^S121A^ on tumor growth indicates that reduced stability of SPRY2 blocks GB tumor growth.

Although inhibition of miR-21 increases the SPRY2 protein in GB cells, inhibition of miR-21 reduces proliferation and invasion of GB cells and reduces tumor growth in intracranial rat and mouse GB xenograft models [85,86,119,120]. Furthermore, miR-21 decreases the SPRY2 protein but enhances the resistance of SWOZ2 human glioma cells to carmustine (BCNU), an alkylating cytostatic for GB treatment. The effect of reduced SPRY2 levels on the increased resistance of SWOZ2 cells to BCNU was confirmed by SPRY2 knockdown [121]. MiR-27a reduces SPRY2 and FOXO3 in U87 GB cells, but the inhibition of miR-27a impairs the proliferation of U87 cells and the tumor growth of U87 cells in subcutaneous mouse tumor xenografts, in which SPRY2 and FOXO3 are increased [92]. Inhibition of miR-27b increases the SPRY2 protein but suppresses cell invasion of U251 cells, and this effect is reversed by SPRY2 knockdown [93]. The contradictory effects of the miRs regulating SPRY2 on GB tumorigenesis compared to the effects of direct SPRY2 regulation may be caused by the fact that miRs such as miR-21 or miR-27a also have targets other than SPRY2 in GB [6,81,92].

SPRY3 mRNA expression in GB is much lower than the expression of SPRY1, SPRY2, or SPRY4 [59]. TCGA datasets concerning the effects of SPRY3 on the survival of GB patients are missing. The overall survival of adult glioma patients with low SPRY3 levels is non-significantly reduced, whereas there is no effect in pediatric glioma patients (Figure 4). SPRY3 protein levels are higher in GB cell lines than in cell lines from lower-grade glioma. Overexpression of SPRY3 increases proliferation and migration of U373 and DBTRG-05 GB cells, whereas downregulation of SPRY3 reduces the growth and the migration velocity of these cells [60].

SPRY4 has been identified as a biomarker gene by single-cell sequencing with differential expression in the different subtypes of glioma [122]. SPRY4 mRNA is reduced in GB compared to adjacent brain tissues, and high expression of SPRY4 is associated with better prognosis of GB patients [97,123]. By contrast, our TCGA data analysis revealed no effect on the overall survival of GB patients with high SPRY4 levels, but the progression-free survival of patients with high SPRY4 levels is reduced (Figure 3). Furthermore, overall survival of adult glioma patients with high SPRY4 levels is strongly reduced (Figure 4A), whereas in pediatric glioma patients, high expression of SPRY4 is associated with a non-significantly better prognosis (Figure 4B). SPRY4 overexpression inhibits proliferation, migration, and invasion of U373 and DBTRG-05 GB cells by reducing ERK activation and matrix metalloproteinase 9 (MMP9) expression [60,123]. MiR-1908 reduces SPRY4, promotes proliferation and invasion of U251 cells, and increases expression of MMP2 and MMP9. Furthermore, miR-1908 is upregulated in GB, and high levels of miR-1908 are strongly associated with shorter survival times in GB patients [97].

### 3.2. Sprouty and Neuroblastoma

**Figure 5:**
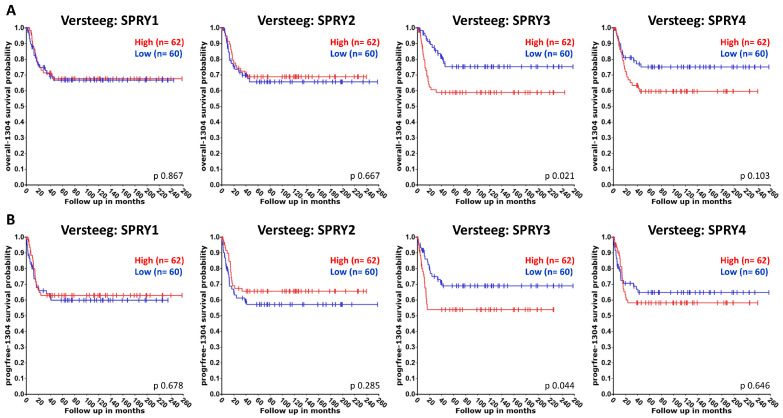
The association of the expression of SPRY1-4 with the overall (**A**) and progression-free (**B**) survival of NB patients was evaluated in Versteeg datasets. Overall and progression-free survival of patients with high SPRY3 and -4 levels are reduced.

**Figure 6:**
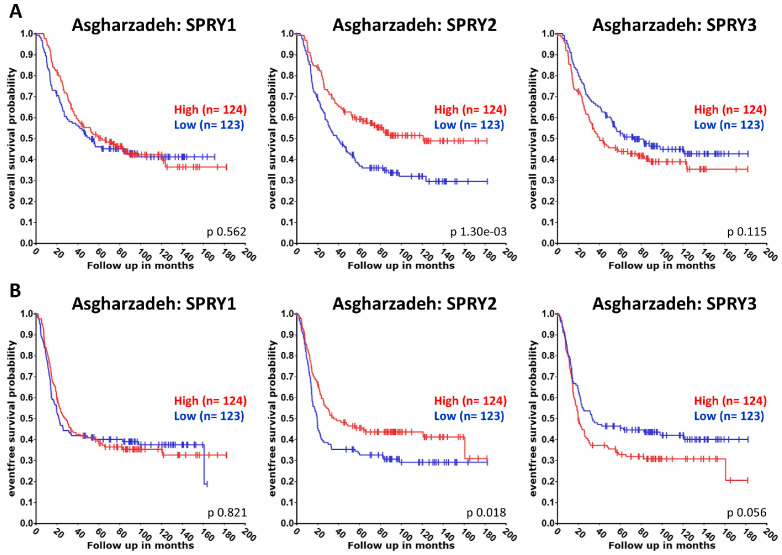
The association of the expression of SPRY1-3 with the overall (**A**) and event-free (**B**) survival of NB patients was evaluated in the Asgharzadeh datasets. Overall and event-free survival of patients with low SPRY2 and high SPRY3 levels is reduced.

**Figure 7:**
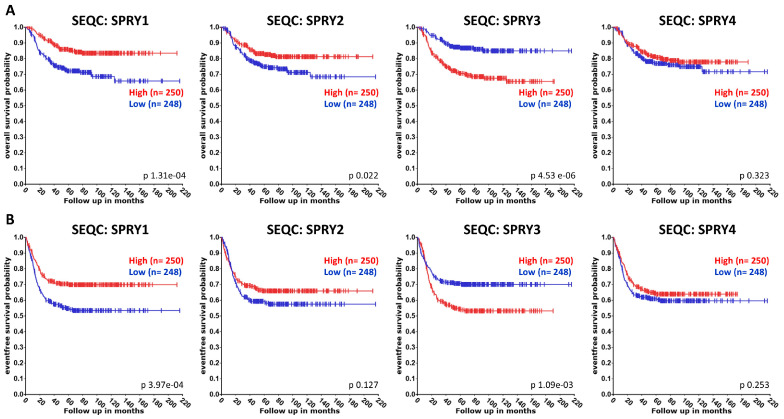
The association of the expression of SPRY1-4 with the overall (**A**) and event-free (**B**) survival of NB patients was evaluated in SEQC datasets. Overall and event-free survival of patients with low SPRY1 and -2 and high SPRY3 levels are reduced.

Publications about the functions of SPRY proteins in NB are summarized below, although, to date, there are only a few publications available. Thus, we also used the R2 platform to compare the association of SPRY1-4 with the overall and event/progression-free survival of NB patients using datasets from Versteeg (NB88 dataset GSE16476 extended with additional cases), Asgharzadeh, and SEQC (GSE62564). Univariate Kaplan–Meier analyses were performed as described above for GB and glioma analyses [114]. The resulting survival curves and *p*-values (obtained via the log-rank test with Bonferroni correction for multiple comparison) were downloaded (Figure 5, Figure 6 and Figure 7).

The Versteeg and Asgharzadeh datasets reveal no difference in the overall or event/progression-free survival of NB patients with high or low SPRY1 levels (Figure 5 and Figure 6). By contrast, SEQC datasets demonstrate that high SPRY1 levels improve overall and event-free survival of NB patients (Figure 7). SPRY1 mRNA was much lower in TGW human NB cells than SPRY2 and SPRY4 mRNA. Thus, the effects of SPRY1 on NB cell proliferation were not further investigated in this study [61]. MiR-124 reduces SPRY1 protein in cortical neurons, whereas it reduces ROCK1 in M17 NB cells [94,95]. MiR-124 inhibits the proliferation of M17 NB cells, whereas it induces neurite outgrowth of undifferentiated NB cells and enhances neurite elongation of differentiated NB cells [94,96].

The Versteeg datasets reveal no difference in the overall survival of patients with high or low SPRY2 levels, but a small, non-significant increase in the progression-free survival of patients with higher SPRY2 levels (Figure 5). The Asgharzadeh and SEQC datasets show an improvement in the overall and event-free survival of NB patients with higher SPRY2 levels (Figure 6 and Figure 7). SPRY2 mRNA and protein are increased by GDNF treatment in NB cells in a biphasic way. Overexpression of SPRY2 inhibits GDNF-induced ERK activation and proliferation of TGW NB cells, whereas dominant-negative SPRY2 enhances their growth [61]. The inhibitory effect of reduced ERK activation on NB proliferation also correlates with the inhibitory role of dendritic cell factor 1 (DCF1), which reduces ERK phosphorylation and proliferation of NB cells [124]. These studies suggest that ERK activation is related to the proliferation of NB cells, and SPRY2, as an inhibitor of the ERK pathway, plays an important role in these mechanisms. Several studies report a tumor-promoting role of miR-21 in the progression of NB [89,125]. MiR-21 decreases SPRY2 in SH-SY5Y NB cells, and the transcription factor FOXO3 reduces miR-21, thereby inhibiting the miR-21-induced reduction of SPRY2. Thus, FOXO3 inhibits the activation of ERK via miR-21/SPRY2, which inhibits the proliferation, migration, and invasion of NB cells. These results were further confirmed by a xenograft tumor model in which FOXO3 overexpression reduced tumor growth in vivo, accompanied by reduced miR-21 expression, elevation of SPRY2, and inhibition of ERK signaling. This inhibitory effect of FOXO3 overexpression on tumor growth was diminished by downregulation of SPRY2, confirming the important interplay between FOXO3, miR-21, and SPRY2 in NB [90].

The Versteeg, Asgharzadeh, and SEQC datasets demonstrate the improved overall and event/progression-free survival of NB patients with low SPRY3 levels (Figure 5, Figure 6 and Figure 7). The mRNA of SPRY3 was much lower in TGW human NB cells than the mRNA of SPRY2 or SPRY4. After GDNF treatment, SPRY3 mRNA was reduced after 2 h but then increased again after 16 h [61]. The SPRY3 protein is much higher in the SK-N-DZ and SK-N-FI NB cell lines than in GB cell lines, independently of the serum levels [60].

Overall and progression-free survival of NB patients with low SPRY4 levels tend to increase in the Versteeg dataset, although the difference is not significant (Figure 5). Asgharzadeh datasets are missing for SPRY4, and the SEQC dataset reveals no difference in the overall or event-free survival of NB patients with high or low SPRY4 levels (Figure 6 and Figure 7). The mRNA and protein of SPRY4 are induced by GDNF in TGW human NB cells but to a lower extent than SPRY2 [61]. SPRY4 protein is not detectable in SK-N-DZ NB cells, but it can be induced by serum in the SK-N-FI NB cells [60].

## 4. Conclusions

**Figure 8:**
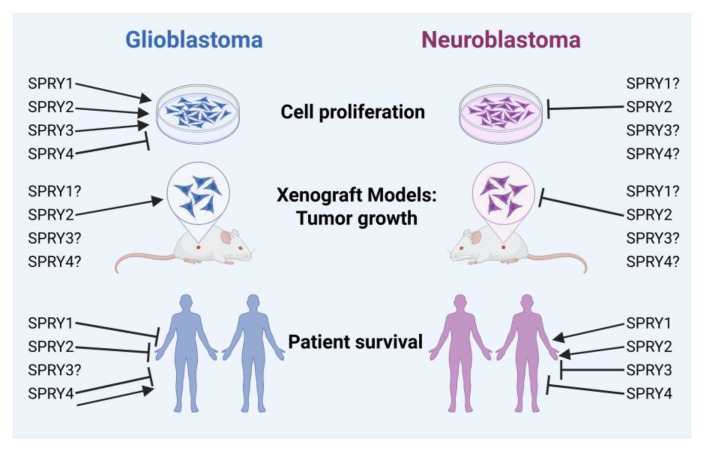
Overview of the effects of SPRY1-4 on the tumorigenesis of GB and NB. Left: High SPRY1 levels increase proliferation of GB cells and reduce survival of GB patients. Increased SPRY2 levels enhance proliferation of GB cells, tumor growth of subcutaneous and intracranial xenografts, and reduce survival of GB patients. SPRY3 enhances proliferation of GB cells, but data concerning the survival of GB patients with high SPRY3 levels are missing. SPRY4 inhibits proliferation of GB cells, but different datasets concerning the effects of SPRY4 on the survival of GB patients are contradictory. Right: Data concerning the effects of SPRY1, -3, and -4 on the cell proliferation of NB cells are lacking. SPRY2 inhibits cell proliferation of NB cells and tumor growth of xenografts. Accordingly, patients with high SPRY2 levels reveal increased survival rates. SPRY1 increases survival of NB patients as well, whereas high SPRY3 or -4 levels reduce the survival of NB patients. Image was created in BioRender. Hausott, B. (2025) https://BioRender.com/q0p3h4p.

The data in this review demonstrate that SPRY1 is an oncogene in GB because high SPRY1 levels are associated with reduced survival of GB and adult glioma patients. The oncogenic role of SPRY1 in GB is also supported by in vitro studies. Several in vitro and in vivo studies demonstrate that SPRY2 is an oncogene in GB as well, and high SPRY2 levels reduce survival of GB and glioma patients. By contrast, the effects of SPRY3 and -4 on the tumorigenesis of GB are not well defined. The TCGA datasets concerning the effects of SPRY3 in GB are lacking, and the survival of adult glioma patients with low SPRY3 levels is non-significantly reduced. However, downregulation of SPRY3 inhibits the growth and migration of GB cells [60]. Progression-free survival of GB patients and overall survival of adult glioma patients with high SPRY4 levels were reduced in our R2 genomics analysis, whereas other studies revealed that high expression of SPRY4 is associated with better prognosis for GB patients [123]. The different effects of SPRY4 in the survival analyses may be dependent on the dataset (sample size, cohort, and analysis method) and the methodology of the survival curve analysis (mean versus median expression cut-off). In vitro studies demonstrate that SPRY4 overexpression inhibits the proliferation and migration of GB cells [60] (Figure 8).

Studies on the effects of SPRY1 on NB proliferation are missing, and the Versteeg and Asgharzadeh datasets reveal no difference in the survival of NB patients with high or low SPRY1 levels. By contrast, SEQC datasets demonstrate that high SPRY1 levels improve survival in NB patients. The Asgharzadeh and SEQC datasets show enhanced survival of patients with high SPRY2 levels. Furthermore, in vitro and in vivo studies confirm that high SPRY2 levels reduce the tumorigenesis of NB. The survival of NB patients with low SPRY3 levels is increased in all three datasets. Although in vitro and in vivo studies about the effects of SPRY3 in NB tumorigenesis are lacking, the survival analysis implicates an oncogenic role for SPRY3 in NB, which has to be further investigated. By contrast, the survival analysis of NB patients with high or low SPRY4 levels reveals no significant differences, and in vitro and in vivo studies are missing (Figure 8).

Together, there is evidence that SPRY1 and -2 are oncogenes in GB, whereas SPRY2 is a tumor suppressor in NB. The opposite effects of SPRY2 in GB and NB may at least be partially explained by its effects on ERK activation. In GB, excessive ERK activation induced by SPRY2 knockdown reduces proliferation, whereas reduced ERK activation caused by SPRY2 overexpression inhibits the proliferation of NB cells. The effects of SPRY3 and -4 in GB and of SPRY1, -3, and -4 in NB are insufficiently investigated, although the survival of NB patients with low SPRY3 levels increased in all datasets. Further in vitro and in vivo studies on the effects of all four SPRY isoforms in tumors of the nervous system are necessary to better define their roles as potential therapeutic targets in the future. In addition, the effects of SPRY proteins on the treatment resistance of GB and NB tumors need to be further evaluated to define their potential for combination therapies with chemotherapeutics or RTK inhibitors. The relevance of SPRY post-translational modifications in tumor progression needs to be explored as well.

Targeting SPRY in the nervous system requires efficient delivery systems. Lipophilic nano-delivery platforms such as liposomal or polymeric nanoparticles mediate transport across the blood–brain barrier (BBB). Furthermore, surface receptor or substrate selectivity of the nano-delivery system influences transport [126]. Transferrin-modified magnetic nanoparticles deliver small interfering RNA (siRNA) to GB across the BBB in mice after tail vein injection and magnetic field application [127]. Intracranial injection of folate-targeted nanocarriers incorporating siRNA was applied to downregulate B-cell lymphoma 2 (BCL-2) in orthotropic glioma in rats [128]. Furthermore, cell-penetrating peptides and chitosan nanocarriers that cross the BBB are used for the regulation of different genes in GB animal models [129,130,131]. Lipid-based nanovesicles and disialoganglioside GD2-coated nanoparticles deliver miRs to NB xenografts after intravenous injection [132,133]. To improve the delivery and efficacy of siRNA to NB xenografts, a bispecific antibody for recognition of EGFR and methoxy polyethylene glycol (PEG) is combined with a PEGylated siRNA lipid nanoparticle, which leads to increased cell targeting against EGFR+ high-risk NB cells [134]. Multifunctional nanoplatforms, such as carbon nanotubes and porous organic frameworks, will be ideal carriers for the next generation of gene drugs. Due to their high loading capacity and multifunctionality, they provide powerful tools for studying the function of SPRY proteins and developing their targeted therapies [135].

## Figures and Tables

**Table 1:** MiRs that regulate SPRY isoforms in glioblastoma (GB) and neuroblastoma (NB) cells and tissue: MiR-21 is deregulated in GB and NB and targets SPRY1 and -2 in these types of tumor cells. MiR-27a and -b target SPRY2 in GB, and miR-124 regulates SPRY1 in NB cells. SPRY4 is regulated by miR-1908, miR-25-5p, and miR-130b-5p in GB cells and neurospheres.

MicroRNA	SPRY Isoform	Cell/Tissue Type	References
MiR-21	SPRY1	Reduction in SPRY1 protein caused by miR-21 correlates with the differentiation of GB-initiating cells.	[87]
MiR-21	SPRY2	Anti-miR-21 increases SPRY2 protein in U87 GB cells, and high miR-21 levels correlate with reduced SPRY2 protein in GB tissue.	[86]
MiR-21	SPRY2	MiR-21 reduces SPRY2 mRNA and protein in SH-SY5Y NB cells and increases their proliferation and migration.	[89,90]
MiR-27a	SPRY2	MiR-27a reduces SPRY2 protein in U87 GB cells.	[92]
MiR-27b	SPRY2	MiR-27b reduces SPRY2 protein in U251 GB cells, and miR-27b inhibition impairs cell migration.	[93]
MiR-124	SPRY1	MiR-124 reduces SPRY1 protein in cortical neurons and inhibits proliferation of M17 NB cells.	[95,96]
MiR-1908	SPRY4	MiR-1908 reduces SPRY4 mRNA and protein in U251 GB cells and increases proliferation.	[97]
MiR-25-5p	SPRY4	Downregulation of miR-25-5p in GB neurospheres increases SPRY4 mRNA.	[98]
MiR-130b-5p	SPRY4	Downregulation of miR-130b-5p in GB neurospheres increases SPRY4 mRNA.	[98]

## Data Availability

All obtained datasets supporting the conclusions of this article are included within the article.

## Abbreviations

The following abbreviations are used in this manuscript:

ALK	Anaplastic lymphoma kinase
BBB	Blood–brain barrier
BCL-2	B-cell lymphoma 2
BCNU	Carmustine
BDNF	Brain-derived neurotrophic factor
C-CBL	C-casitas b-lineage lymphoma
CNS	Central nervous system
CRD	Cysteine-rich domain
DCF1	Dendritic cell factor 1
EGFR	Epidermal growth factor receptor
ERBB3	Erb-B2 receptor tyrosine kinase 3
ERK	Extracellular signal-regulated kinase
EST	Expressed sequence tag
FGFR	Fibroblast growth factor receptor
FOXO3	Forkhead box O3
GB	Glioblastoma
GBM	Glioblastoma multiforme
GDNF	Glial cell-line-derived neurotrophic factor
GRIN	G protein-regulated inducer of neurite outgrowth
IDH	Isocitrate dehydrogenase
IGF1R	Insulin-like growth factor 1 receptor
IGFBP2	Insulin-like growth factor binding protein-2
INRGSS	International Neuroblastoma Risk Group Staging System
IR	Insulin receptor
JNK	Jun N-terminal kinase
MAPK	Mitogen-activated protein kinase
MEK	Mitogen-activated protein kinase kinase
MET	Hepatocyte growth factor receptor
MiR	MicroRNA
MKP	Mitogen-activated protein kinase phosphatase
Mnk1	Mitogen-activated protein kinase-interacting kinase 1
MMP	Matrix metalloproteinase
NB	Neuroblastoma
NEDD4	Neural precursor cell-expressed developmentally downregulated protein 4
NF-κB	Nuclear factor kappa B
NGF	Nerve growth factor
NT3/4	Neurotrophin-3/4
PEG	Polyethylene glycol
PDGFR	Platelet-derived growth factor receptor
PI3K	Phosphoinositide 3-kinase
PKB/Akt	Protein kinase B
PLCγ1	Phospholipase Cγ1
PNS	Peripheral nervous system
RAS	Rat sarcoma
RTK	Receptor tyrosine kinase
SCA	Segmental chromosome aberrations
siRNA	Small interfering RNA
SIAH2	Seven in absentia homolog 2
SPRY	Sprouty
SRM	Serine-rich motif
STAT3	Signal transducer and activator of transcription 3
TACC	Transforming acidic coiled-coil
TCGA	The Cancer Genome Atlas
TERT	Telomerase reverse transcriptase
Trk	Tropomyosin receptor kinases
VEGF	Vascular endothelial growth factor
WHO	World Health Organization
